# Cytomegalovirus Retinitis Complicated With Rhegmatogenous Retinal Detachment in a Non-HIV Patient With Pure Red Cell Aplasia

**DOI:** 10.7759/cureus.92998

**Published:** 2025-09-23

**Authors:** Chern Meng Tan, Mat Saad Noram

**Affiliations:** 1 Department of Ophthalmology, Hospital Tuanku Jaafar, Seremban, MYS

**Keywords:** cmv retinitis, immunosuppressed, non-hiv patient, rhegmatogenous retinal detachment (rrd), thymoma-associated pure red cell aplasia

## Abstract

Cytomegalovirus (CMV) is a double-stranded DNA virus belonging to the Herpesviridae family. It can cause CMV retinitis, an opportunistic infection in immunocompromised patients. This is a sight-threatening condition that can be further complicated by rhegmatogenous retinal detachment (RRD). In this case report, we described a 57-year-old gentleman with underlying pure red cell aplasia on ciclosporin who presented with blurred vision in both eyes. The retinal findings showed frosted branch angiitis, granular appearance, and scattered retinal hemorrhages. Confirmation of CMV retinitis was done using polymerase chain reaction of the vitreous sample and CMV serum serology. The patient was treated with intravenous ganciclovir and intravitreal ganciclovir in the right eye. He developed CMV-related RRD in the left eye, for which he underwent retinal detachment surgery with a good anatomical outcome, but developed a significant cataract. Early recognition and treatment of CMV retinitis can improve visual outcomes. Physicians and patients on any form of immunosuppression should be familiar with the symptoms of CMV retinitis and retinal detachment.

## Introduction

Cytomegalovirus (CMV) is a double-stranded DNA virus belonging to the Herpesviridae family. The estimated CMV seroprevalence is 83% in the general population [1]. Healthy individuals are usually asymptomatic, but in immunocompromised populations, such as those with AIDS or those on systemic immunosuppressants, CMV can cause opportunistic infections [2]. CMV retinitis is a sight-threatening condition, especially located in zone one or complicated by retinal detachment [3,4]. Based on the Standardization of Uveitis Nomenclature Working Group criteria, clinical diagnosis of CMV retinitis is established based on findings of necrotizing retinitis, which can manifest as wedge-shaped, hemorrhagic, or granular lesions with indistinct borders in an immunocompromised host, irrespective of laboratory evidence of systemic or intraocular CMV infection [5]. In this case, we highlight the importance of a multidisciplinary approach in the management of CMV retinitis and its complication, rhegmatogenous retinal detachment (RRD).

## Case presentation

**Figure 1:**
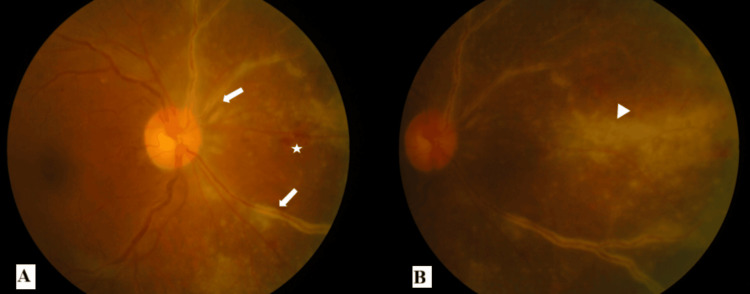
Fundus photographs of the right eye at presentation (A) Presence of frosted branch angiitis (white arrow) over the superonasal and inferonasal arcades originating from the optic disc (zone one) with scattered retinal hemorrhages (star). (B) Presence of retinitis (white arrowhead) at the nasal retina

**Figure 2:**
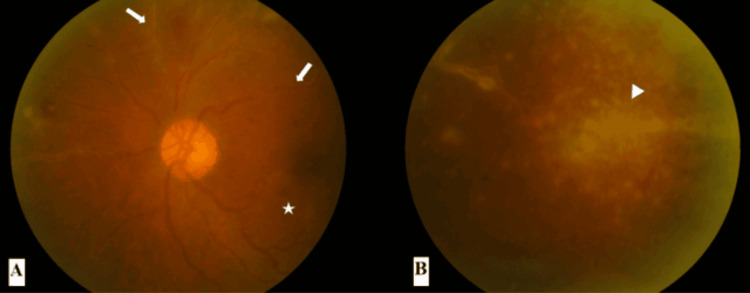
Fundus photographs of the left eye at presentation (A) Presence of frosted branch angiitis (white arrows) over the superonasal and superotemporal arcades (zone two), retinal veins tortuosity, and scattered retinal hemorrhages (star). (B) Granular appearance (white arrowhead) of the retina in the mid-peripheral superotemporal quadrant

A 57-year-old gentleman presented with blurred vision in both eyes for three weeks. It was described as “watermarks on the windscreen.” At presentation, there was no relative afferent pupillary defect (RAPD). The best corrected visual acuity in bilateral eyes was 6/9. There were keratic precipitates in bilateral eyes with anterior chamber cells of 1+ in the right eye and 2+ in the left eye. The intraocular pressure in bilateral eyes was 14 mmHg. The fundus of the right eye showed frosted branch angiitis over the superonasal and inferonasal arcades originating from the optic disc (zone one) with scattered retinal hemorrhages and retinitis at the nasal retina (Figures 1A, 1B). The fundus of the left eye showed a granular appearance in the superotemporal mid-peripheral retina and frosted branch angiitis over the superonasal and superotemporal arcades (zone two) with scattered retinal hemorrhages (Figures 2A, 2B). There was no vitritis in either eye, and both optic discs were not swollen.

His significant medical history was thymoma-associated red cell aplasia on oral ciclosporin 150 mg twice daily (BD) under the Hematology Department follow-up. He reported the ocular symptom one month after the initiation of oral ciclosporin. His relevant past surgical history was a thymectomy for thymoma.

He was clinically diagnosed with bilateral CMV retinitis based on the fundoscopic examination (right eye zone one, left eye zone two) with an underlying immunocompromised state. He was admitted to the ward and planned for induction of intravenous ganciclovir and right eye intravitreal (IVT) ganciclovir. Baseline bloods were screened. His full blood count showed bicytopenia with a hemoglobin of 7.6 g/L (normal range: 13-17 g/L) and a white cell count of 1.8 x 10^9^/L (normal range: 4-10 x 10^9^/L). His absolute neutrophil count (ANC) was 0.32 x 10^3^/uL (normal range: 2-7 x 10^3/^uL). The platelet count was normal. Renal profile and liver function test were both normal. His serum CMV IgM was nonreactive, and IgG was reactive with a reading of 2,198 IU/mL (normal range: 600-1,600 IU/mL). Biohazard screenings for syphilis, hepatitis B, hepatitis C, and human immunodeficiency virus were all nonreactive. Hematology and infectious disease teams were consulted before induction of intravenous ganciclovir at 5 mg/kg BD. He was given subcutaneous Neupogen 300 mg Nocte twice per week to increase the white cell count while oral ciclosporin was maintained at 150 mg BD. Gutt dexamethasone BD was prescribed for bilateral eyes.

**Figure 3:**
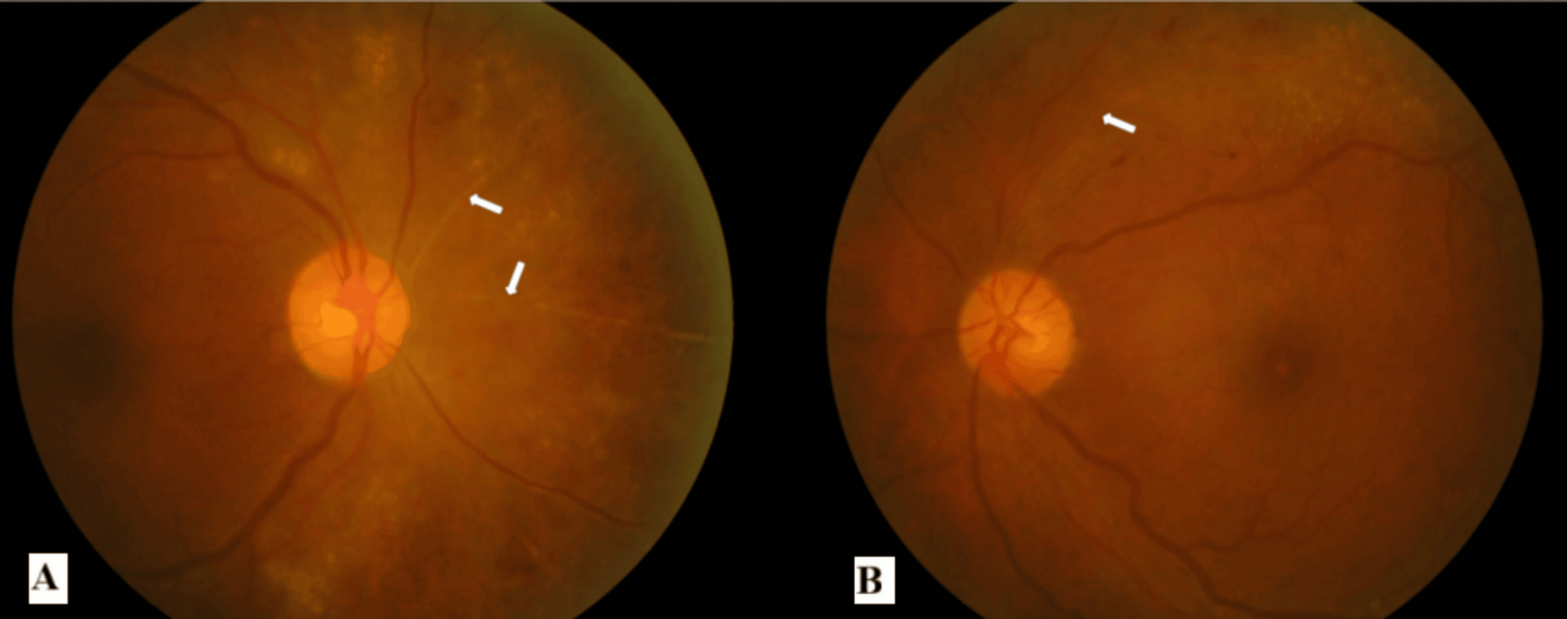
Fundus photographs of bilateral eyes after treatment (A) The right eye showed sclerosed vessels over the nasal quadrant (white arrows). (B) The left eye showed a sclerosed vessel over the super-temporal arcade (white arrow)

Right eye vitreous tap was performed and sent for CMV polymerase chain reaction (PCR). He was commenced on biweekly right eye IVT ganciclovir 2 mg/0.1 mL. Laboratory investigation revealed a positive PCR for CMV, confirming the diagnosis of CMV retinitis. Upon completion of three weeks of IV ganciclovir and four doses of right eye IVT ganciclovir, the bilateral eye vision was 6/9. Bilateral eye retinitis resolved with scarring, leaving behind sclerosed vessels with minimal retinal hemorrhages (Figures 3A, 3B). The infectious disease team started the patient on maintenance therapy with oral valganciclovir 900 mg once daily for 10 weeks after he completed three weeks of induction therapy. His ANC was 2.17 x 103/uL (normal range: 2-7 x 10^3^/uL) upon discharge. He was given subcutaneous Neupogen every other day until the completion of oral valganciclovir.

He was monitored monthly with dilated fundus examination, which showed pigmentary changes and scarring at previous retinitis locations. Bilateral eye optical coherence tomography of the macula was normal. Five months following initial diagnosis, he presented to the eye casualty with a two-week history of left superior visual field defect. Left eye RAPD was positive with visual acuity of 6/36; anterior segment examination was normal. The fundus of the left eye showed inferior RRD from 3 to 8 o'clock, detached macula, presence of proliferative vitreoretinopathy (PVR) grade C at 6 o'clock, and retinal break at 8 o'clock. His right eye vision was 6/9. There were no signs of CMV reactivation in the bilateral eyes. He was planned for left eye retinal detachment surgery and comanaged with the hematology team for the optimization of blood parameters before the surgery.

**Figure 4:**
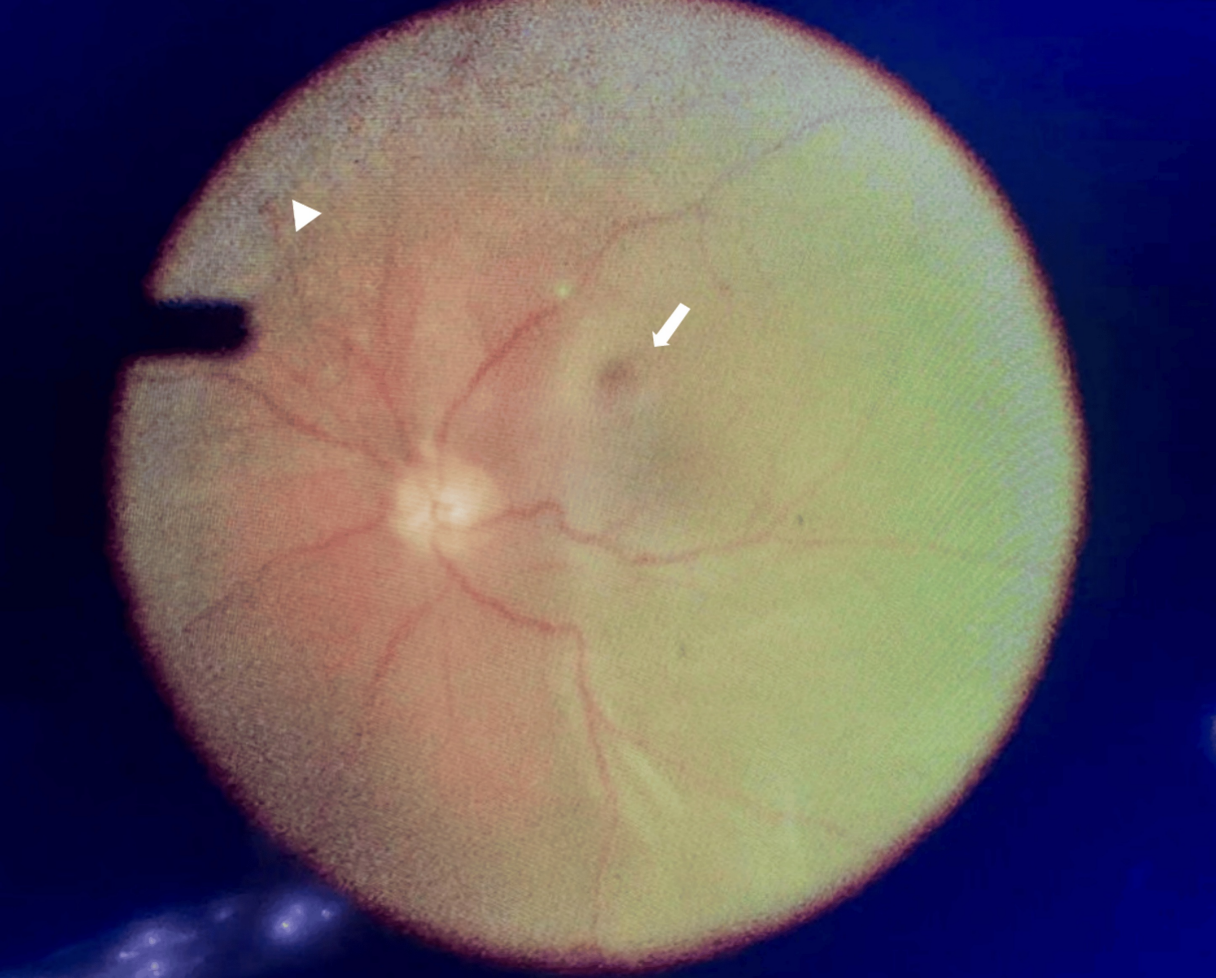
Intraoperative findings of the left eye Presence of subtotal retinal detachment from 2 to 10 o'clock (shallow detachment from 6 to 10 o'clock), macula was detached (white arrow), retinal break identified at 8 o'clock, and pigmentary changes (white arrowhead) over the superior one third of the retina

He underwent left eye scleral buckle, pars plana vitrectomy, 360° endolaser, and silicone oil tamponade under general anesthesia. Intraoperatively, there was subtotal retinal detachment from 2 to 10 o'clock (shallow detachment from 6 to 10 o'clock), the macula was detached, a retinal break was identified at 8 o'clock, and pigmentary changes over the superior one-third of the retina (Figure 4). Two months postoperatively, the retina of the left eye was flat. Unaided visual acuity for the left eye was 1/60 secondary to a visually significant cataract and a silicone oil-filled globe. The right eye vision remained at 6/9. There were no signs of CMV reactivation in both eyes. Six months after the surgery, he was diagnosed with Good's syndrome based on the history of thymoma, low CD4+ T cell count of 30 cells/mm³ (normal range: 500-1,500 cells/mm³), and recurrent respiratory tract infections. Eventually, he succumbed to the disease.

## Discussion

Pure red cell aplasia (PRCA) is a hematological condition affecting the red cell lineage, characterized by severe normochromic and normocytic anemia, marked reticulocytopenia, and absence of erythroblasts [6]. This patient was diagnosed with secondary acquired PRCA associated with thymoma, for which he underwent thymectomy as part of the treatment. The mainstay of the treatment for PRCA is immunosuppression. He was given oral ciclosporin, the most effective immunosuppressant, with a more than 75% response rate according to the literature [6]. However, he developed CMV retinitis, which is an opportunistic infection secondary to his immunocompromised state.

Drug-to-drug interactions between ciclosporin and ganciclovir can potentially lead to adverse drug reactions such as acute kidney injury and further bone marrow suppression. In this patient, the challenges we faced were that he had bicytopenia before the initiation of ganciclovir. His hemoglobin was low due to underlying red cell aplasia, and his white cell count was low due to ciclosporin. We managed with Hematology to optimize his white cell counts using subcutaneous Neupogen during concurrent treatment with ciclosporin and ganciclovir.

This patient was not given IVT ganciclovir in the left eye as the CMV retinitis was located at zone two. Five months after the diagnosis of CMV retinitis, he developed RRD in the left eye. A retrospective study conducted by Young et al reported that the rate of retinal detachment in CMV retinitis is 14-fold higher in patients treated with intravenous therapy compared to IVT therapy [4]. The risk is higher if there is more than or equal to 25% of retinal area involvement. This could be explained by the fact that the infected necrotic retina is replaced by glial tissue, which is poorly adherent to the underlying retinal pigment epithelium and choroid [4]. Traction to such an area at the edges by the attached posterior hyaloid might cause retinal tears and subsequently lead to RRD [4]. It is postulated that IVT injections can effectively control the retinitis and cause posterior vitreous detachment, resulting in a lower rate of retinal detachment [4].

A study published by Wong et al. reported that anatomical success rates after retinal detachment surgery for CMV-related retinal detachment were lower due to multiple breaks located in the necrotic retina, which caused difficulty in identifying and sealing the breaks [7]. In CMV retinitis-related retinal detachment surgery, the anatomical success rates reported in the current literature are 70%-84% [7], which is lower than those of primary RRD surgery, with anatomical success rates of 72%-92% [8,9]. Factors that contributed to better anatomical outcomes, as reported by Wong et al, were lesions with less than 50% retinal area involvement, the use of 23-gauge vitrectomy with a wide-field viewing system, and the absence of PVR [7]. Good anatomical outcomes were also observed in this case due to the favorable factors mentioned. Regarding the visual prognosis of treated CMV retinitis between HIV and non-HIV patients, the current literature did not favor either group; however, both studies consistently concluded that the factor that contributed to poor visual outcomes was retinal detachment [10,11].

## Conclusions

CMV retinitis and retinal detachment are sight threatening conditions which could result in permanent visual loss and affect the quality of life. Early recognition and prompt treatment can improve the visual outcomes. The multidisciplinary approach remained the cornerstone in the management of CMV retinitis in non-HIV patients. Screening for CMV retinitis is recommended in non-HIV patients who are taking high dose immunosuppressants however there is no consensus on screening guidelines as for HIV patients. Therefore, physicians should be familiar with the symptoms of CMV retinitis and retinal detachment and refer promptly to the Ophthalmology team if there is a high index of suspicion. Patients who are treated for CMV retinitis shall be educated on the red flags of retinal detachment.
